# Occlusion and Its Role in the Long-Term Success of Dental Restorations: A Literature Review

**DOI:** 10.7759/cureus.73195

**Published:** 2024-11-07

**Authors:** Amirah F Aldowish, Munirah N Alsubaie, Sundees S Alabdulrazzaq, Deem B Alsaykhan, Amal K Alamri, Lama M Alhatem, Joud F Algoufi, Saud S Alayed, Sara S Aljadani, Ashwaq M Alashjai, Abdulrahman S Alamari

**Affiliations:** 1 Dentistry, King Saud bin Abdulaziz University for Health Sciences, Riyadh, SAU; 2 Dentistry, Nakaa Medical Polyclinic, AlUla, SAU; 3 Dentistry, King Saud University, Riyadh, SAU; 4 General Dentistry, King Abdulaziz University, Jeddah, SAU; 5 General Dentistry, Riyadh Elm University, Riyadh, SAU; 6 Family Dentistry, Ministry of Health, Riyadh, SAU

**Keywords:** dental prostheses, occlusion, parafunctional habits, restoration longevity, temporomandibular disorders

## Abstract

Occlusion plays a fundamental role in the long-term success of dental restorations by influencing both their functional stability and durability. This review explores the occlusal considerations for various restorative modalities, including fixed and removable prostheses, implant-supported restorations, and adhesive restorations. Special attention is given to the biomechanical principles involved, such as force distribution, stress management, and the role of occlusion in temporomandibular joint health. Diagnostic tools, including traditional and digital techniques such as T-Scan and OccluSense, are discussed to highlight their relevance in detecting occlusal disharmony. Additionally, the review examines the effects of parafunctional habits on restoration longevity and the influence of occlusal trauma on prosthetic outcomes. Despite advances in materials and technology, achieving functional occlusion remains essential in minimizing complications and ensuring patient comfort. This review underscores the need for proper occlusal analysis during treatment planning to enhance clinical outcomes and extend the lifespan of dental restorations.

## Introduction and background

Occlusion remains one of the most debated topics in modern dentistry. Early ideas of occlusion were primarily mechanical, and practitioners focused on achieving harmonious tooth alignment for effective mastication [1]. However, the understanding of occlusion has expanded to include not only mechanical aspects but also the dynamic, functional, and neuromuscular interactions between teeth, muscles, and temporomandibular joints (TMJ) while chewing, speaking, and at rest. This broader perspective has had a significant impact on how dental practitioners approach occlusion-related considerations in treatment planning and restorations [1-5].

In 1898, Edward H. Angle introduced the concept of ideal occlusion as a key goal for orthodontic treatment [2]. Angle’s theory, based on empirical observations, was guided by a skull he affectionately named “Old Glory,” which represented his vision of optimal occlusion for patients [2]. His ideas laid the groundwork for subsequent theories, which were further refined throughout the 20th century. In 1926, McCollum and Stuart advanced these concepts by developing gnathology, which emphasized precise coordination between the TMJ and occlusal surfaces to achieve optimal function [3]. Their work continues to influence modern restorative and prosthodontic practices.

An anthropological perspective provides additional insight into occlusion. Tooth wear, often viewed as detrimental in contemporary dentistry, was historically a natural adaptation. Early humans exhibited significant tooth wear due to rough diets, yet their dentition remained functional and well-suited to survival [3-7]. In line with these observations, Ogawa et al. argued that balancing contacts during mastication might be more beneficial than previously believed, challenging the conventional emphasis on steep posterior guidance and tight incisal contacts [4]. Furthermore, muscle coordination neglect in earlier occlusal designs contributed to clinical failures, underscoring the need to consider both mechanical alignment and functional harmony [4,6].

In 1958, D’Amico introduced the concept of canine-guided occlusion to distribute occlusal forces more efficiently [5]. Although this approach aimed to protect the posterior teeth, it remains controversial due to its potential impact on the TMJ, with some studies suggesting it may contribute to temporomandibular disorders (TMDs) [6]. This ongoing debate highlights the complexity of achieving a functional occlusion that promotes both durability and patient comfort.

The Glossary of Prosthodontic Terms (GPT-10) defines occlusion as the static relationship between the incising or masticating surfaces of the maxillary and mandibular teeth [7]. However, this definition only captures one aspect of occlusion. In practice, occlusion encompasses dynamic activities such as mastication, swallowing, and bruxism, all of which subject dental restorations to continuous forces throughout their lifespan. When occlusal discrepancies go unaddressed, they often result in restorative failures and manifest as fractures, wear, periodontal complications, or exacerbated TMD [8,9]. According to Goldstein, the longevity of dental restorations depends on multiple factors, such as the materials used, patient-related factors, and dentist-related factors [10]. Gordan et al. attribute the success of restorations to the quality at the time of placement, the type and size of the restoration, the material used, and the clinician’s ability to diagnose secondary caries [11].

A thorough understanding of occlusal relationships is essential to reduce the likelihood of long-term complications. Clinicians equipped with this knowledge can better predict how restorations will behave under functional stress and incorporate occlusal adjustments as part of routine examinations and procedures. By exploring historical perspectives, current concepts, and clinical implications, this review aims to provide a comprehensive understanding of how occlusion plays a pivotal role in the long-term outcomes of dental restorations.

## Review

Materials and methods

This narrative review was conducted to explore the multifaceted role of occlusion in the long-term success of dental restorations, discussing historical context, biomechanical principles, clinical diagnostic methods, and prosthodontic techniques. Key themes include occlusal considerations for fixed and removable prostheses, implant-supported restorations, adhesive restorations, and the impact of occlusal trauma and parafunctional habits on prosthetic longevity.

Literature Search Strategy

A broad literature search was conducted using databases including PubMed, Google Scholar, and Scopus to identify studies that address occlusion in dentistry, occlusal concepts in prosthodontics, temporomandibular joint health, and the influence of occlusal trauma on restorations. Search terms included combinations of “occlusion in dentistry”, “dental restorations and occlusion”, “fixed prosthodontics occlusion”, “implant occlusion”, “occlusal trauma”, and “parafunctional habits in prosthodontics”.

The inclusion criteria focused on peer-reviewed articles, review papers, and clinical studies that address occlusal concepts relevant to prosthodontic restorations, implant stability, and diagnostic methods. Specifically, studies discussing mutually protected occlusion, group function, occlusal schemes for complete dentures, and parafunctional habits impacting occlusal stability were prioritized. Articles and textbooks published primarily within the last 30 years were included, allowing for the integration of select seminal studies by foundational figures such as Angle, D’Amico, and McCollum to provide historical context.

The exclusion criteria filtered out non-English articles, inaccessible studies, and reports lacking substantial data on clinical occlusal adjustments and restorative outcomes. Case reports, editorials, and studies focused solely on orthodontic or non-restorative occlusal treatments were also excluded to maintain the review’s focus on prosthodontic and restorative occlusal considerations.

Types of restorations and their occlusal considerations

In restorative dentistry, occlusion plays a pivotal role in the success of any prosthetic treatment, whether it involves a tooth surface restoration, a crown, a tooth or implant-supported prosthesis, or a tissue-supported prosthesis. While a harmonious occlusal relationship will ensure stability, comfort, and function, inadequate occlusion can result in numerous complications. By having a comprehensive understanding of occlusion during treatment planning, practitioners can achieve optimal outcomes and prevent complications [11]. The latter may include pain or discomfort, tooth mobility, progressive tooth wear, fractured teeth or restorations, and TMJ dysfunction [12]. Notably, occlusal trauma resulting from these issues can further aggravate periodontal conditions, compromising not only the restoration but also the health of the supporting structures.

Bite force varies significantly depending on the location in the oral cavity and therefore influences the design and material selection of prosthesis. Maximum bite force values have been reported to be 390-⁠800 N at the first and second molars, approximately 288 N in the bicuspid region, 208 N at the canine area, and 155 N in the incisor region [13]. This variability emphasizes the need for careful treatment planning, especially in multi-unit restorations and implant-supported prostheses, where overloading can lead to mechanical failure. If a single tooth encounters premature contact during mandibular excursions, an unbalanced force distribution is created, which can result in occlusal trauma [14,15]. Such trauma may manifest as wear facets, enamel fractures, and secondary tooth migration, further disrupting the occlusal scheme. Mechanoreceptors located in the periodontal ligament play a crucial role in sensing these load discrepancies by providing critical sensory feedback through the trigeminal nerve to modulate and protect the occlusal system [16]. Conversely, force distribution becomes more challenging in implant-supported restorations without a periodontal ligament, and meticulous occlusal planning is needed to prevent mechanical overload.

Stress distribution across the dental arch varies according to the region and type of occlusal contact. In maximum intercuspation, the anterior region typically experiences compressive and shear stresses, particularly at the junction between the clinical crown and root. This area bears tensile stress and compression during functional and excursive mandibular movements. Incisal angles experience tensile and shear stresses under normal occlusion, while significant compressive stresses are present in edge-to-edge occlusion, which can increase the risk of fractures if not properly managed [13]. In the posterior region, the cusp tips on the functional side are primarily subject to compressive stress, while the axial angles endure tensile and shear stress on the non-functional side [13]. These differences in stress patterns highlight the biomechanical complexity of the dental arch and underscore the importance of precision in restorative dentistry. Furthermore, unaddressed stress imbalances may contribute to non-carious cervical lesions and marginal fractures, particularly in the cervical regions of anterior teeth and cusp inclines of posterior teeth.

Accurate diagnosis is a crucial first step before initiating any form of restorative treatment. Comprehensive intraoral and extraoral assessments, combined with detailed patient history, are essential to reach an accurate diagnosis and avoid unnecessary procedures [17,18]. The extraoral assessment should include evaluations for any facial asymmetry and restricted mouth opening (typically considered restricted if less than 35 mm) [19]. It should also include an examination of the TMJ for pain or tenderness upon palpation of the joint or associated muscles of mastication, as well as abnormal sounds such as clicking or crepitus [20]. The presence of restricted mandibular movement or deviation during mouth opening may indicate underlying TMJ dysfunction or occlusal disharmony, necessitating further diagnostic investigations, such as panoramic radiographs or cone beam computed tomography (CBCT).

Intraoral assessments should involve an evaluation of both hard and soft tissues. Signs of wear, such as attrition and abrasion, scalloping of the tongue, periodontal weakening, fremitus, and fracture lines on teeth or restorations should be carefully documented, as these may indicate underlying occlusal disharmony [19,20]. Similarly, the assessment should evaluate for fremitus or the palpable movement of teeth during occlusion, as it is a key clinical marker of occlusal overload. The clinician must also assess the periodontal status, as progressive wear and occlusal trauma may compromise the health of the supporting tissues and accelerate bone loss and tooth mobility. Integrating digital tools, such as intraoral scanners, can enhance diagnostic precision by providing 3D occlusal mappings. These will help clinicians identify subtle discrepancies that may not be evident through visual or manual inspection.

Occlusal considerations in fixed prosthodontics

A proper diagnosis and treatment plan can be formulated after obtaining a patient history, performing a clinical examination, and analyzing the articulated casts. When planning for fixed prostheses, clinicians should take into account several occlusal principles to achieve long-term success. The most commonly considered principles are mutually protected occlusion and group function occlusion [21].

Mutually protected occlusion is based on the concept that there should be light occlusal contacts on the anterior teeth in the intercuspal position (ICP), with the anterior teeth being protected by the posterior teeth during occlusion. In this occlusal scheme, retruded contact position (RCP) and ICP are coincident, and excursive movements are guided by the anterior teeth. These movements help disclude and protect the posterior teeth from non-axial forces, which can otherwise contribute to premature wear or restorative failure [12,21]. The posterior teeth are therefore protected from static occlusion, while the anterior teeth bear the dynamic forces during activities such as chewing and grinding. Mutually protected occlusion is particularly beneficial in preventing damage to the posterior teeth, as it minimizes the impact of lateral forces on restorations, helping to prevent fractures and excessive wear over time. However, this scheme may be less effective in patients with parafunctional habits, such as bruxism, as excessive lateral forces could lead to structural failures.

Group function occlusion differs in that lateral excursion is guided by multiple teeth, including both the anterior and posterior teeth. Group function, as defined by the Glossary of Prosthodontic Terms (GPT-10), refers to multiple contact relations between maxillary and mandibular teeth during lateral movements on the working side, where several teeth act as a group to distribute occlusal forces evenly [7]. This even distribution of forces helps prevent localized overloading, reducing the likelihood of fractures or failures in individual teeth. The group function is particularly recommended in cases with compromised anterior guidance, such as in patients with worn anterior teeth or when restoring posterior teeth in elderly patients, where long-term stability is a priority.

Various studies have analyzed the efficacy of both group function and canine-guided occlusion in natural and artificial dentitions. For example, Belser and Hannam found no significant difference in muscle coordination between group function occlusion and canine-guided occlusion [22]. In contrast, Jemt et al. conducted a study on chewing patterns and mandibular movements, reporting that participants found group function occlusion more comfortable than canine-guided occlusion [23]. Interestingly, the study also demonstrated that mandibular speed during the chewing cycle was greater with canine-guided occlusion than with group function [23]. However, Salsench et al. observed that canine-guided occlusion resulted in a longer masticatory cycle, possibly due to the increased effort required to maintain stable canine guidance during lateral movements [24].

Miralles concluded in a review that there is no clear evidence to suggest one occlusal scheme is superior to the other in terms of patient comfort or the longevity of restorations, as outcomes are often influenced by factors such as parafunctional habits and material properties [25]. Overall, there is no definitive evidence supporting the superiority of either occlusal scheme, and both may be suitable depending on the clinical situation [12,21]. Some literature suggests that canine-guided occlusion or mutually protected lateral excursions may offer certain advantages: the canine’s long, wide roots and its position as a “cornerstone” of the dental arch make it well-suited to absorb lateral forces during excursions [26]. Furthermore, the robust anatomy of the canines makes them ideal for protecting posterior restorations from damaging lateral forces, especially in patients with deep overbites or anterior restorations.

When performing occlusal corrections using fixed prostheses, clinicians commonly adopt either the conformative approach or the reorganized approach [27]. The conformative approach is typically used when the existing occlusal vertical dimension or maximum intercuspal position (MIP) does not need changing. In this approach, the goal is to preserve the current occlusal scheme without making significant changes to the occlusal relationships of the remaining teeth. Davies et al. described the EDEC principle to guide the conformative approach, where E stands for examining the preoperative occlusion, D for designing the restoration, E for executing the design, and C for checking that the restoration adds to but does not alter the occlusion. This approach is suitable for cases involving tooth surface restorations, single missing tooth rehabilitation, or multiple missing teeth that do not require changes to the vertical dimension or the maximal intercuspal position [27]. Advantages include better patient comfort and fewer postoperative adjustments, as it aligns restorations with the patient’s habitual occlusion.

The reorganized approach, on the other hand, is used when the existing intercuspal position and/or vertical dimension at occlusion are not favorable for the long-term success of the treatment [28]. This approach seeks to restore the occlusal relationship in centric relation (CR), the most stable and repeatable position of the TMJ [28]. CR is crucial not only for achieving long-term functional stability but also for reducing the risk of TMJ dysfunction. This position is determined using techniques such as bimanual manipulation, as described by Dawson [9], along with the aid of deprogramming devices such as the Lucia Jig [29]. After establishing the CR position and mounting the casts accordingly, the practitioner formulates a detailed treatment plan, which includes diagnostic wax-ups, mock preparations of the casts, stent fabrication at the planned vertical dimension, and provisionalization. Before proceeding with the definitive restoration, it is crucial that the patient is comfortable and satisfied with the function and esthetics of the provisional restorations [28]. Ensuring functional harmony and patient acceptance during the provisional phase reduces the need for postoperative adjustments and contributes to better long-term outcomes.

After the final prosthesis is fabricated, occlusal equilibration may be necessary to ensure proper occlusal contacts and eliminate any high points or interferences that could compromise the long-term success of the restoration. This process involves careful adjustment of the occlusal surfaces to optimize force distribution and minimize the risk of restorative failures. In certain cases, adjustments may be needed even months after placement to account for minor changes in occlusal dynamics, particularly in complex rehabilitations or full-mouth reconstructions.

Occlusal considerations in removable prosthodontics

Complete Dentures

Edentulism is a significant health concern among the elderly population worldwide [30]. The loss of natural teeth affects both oral function and quality of life and increases dependency on removable prostheses. Research has consistently demonstrated that patient satisfaction with complete dentures is largely dependent on two critical factors: the retention of the dentures and their occlusal stability [31]. Achieving optimal occlusion is particularly essential in the elderly population, as patients often present with systemic health challenges, reduced neuromuscular coordination, and compromised oral anatomy. Accordingly, complete denture prosthodontics use various occlusal schemes, and each has its advantages, disadvantages, and specific indications. The most common complete denture occlusal schemes include neurocentric occlusion, lingualized occlusion, non-anatomical occlusion, and balanced occlusion [32].

DeVan first proposed neurocentric occlusion, which is characterized by a flat occlusal surface with no medial or lateral inclinations, directing all occlusal forces centrally onto the posterior teeth [33]. By eliminating eccentric interferences and simplifying occlusal management, especially in cases of class II and class III ridge relations, this scheme provides better control over force distribution and reduces the risk of lateral dislodgement [30-33]. However, neurocentric occlusion compromises esthetics and masticatory efficiency due to the flat occlusal table, which may limit the patient’s ability to grind food effectively. While it promotes functional stability, patient education is needed to manage expectations, especially in terms of reduced chewing performance compared to other occlusal designs.

Non-anatomical occlusion, also called the monoplane occlusal scheme, uses non-anatomical or zero-degree teeth to create a flat surface-to-surface contact in occlusion. This scheme offers freedom of movement in both centric and eccentric positions, providing flexibility and comfort to patients with compromised neuromuscular coordination, such as those with Parkinson’s disease [34]. Its lack of cuspal inclines minimizes horizontal forces and thus reduces the chances of denture displacement. However, as with neurocentric occlusion, this scheme sacrifices masticatory efficiency and esthetics, making it less favorable for patients who prioritize function and appearance. In cases where stability is prioritized over esthetics, such as for institutionalized patients, monoplane occlusion can still be a practical choice.

Lingualized occlusion, introduced by Payne in 1941, involves the palatal cusps of the maxillary posterior teeth occluding against the occlusal surfaces of the mandibular teeth [35]. This design ensures that only the lingual cusps engage during function, eliminating eccentric interferences while maintaining efficient contact [34,36]. The reduced lateral stresses enhance denture stability and increase the effectiveness of lingualized occlusion, particularly in complex rehabilitative cases such as maxillectomies or mandibulectomies. Its superior masticatory efficiency makes it suitable for implant-supported prostheses, where stability and function are critical for patient comfort [36]. Additionally, it ensures smooth gliding during functional movements, reducing the likelihood of occlusal trauma.

Balanced occlusion, as defined by the Glossary of Prosthodontic Terms (GPT-10), is the simultaneous bilateral occlusal contact of anterior and posterior teeth during excursive movements [7]. This occlusal scheme provides superior stability, esthetics, and masticatory efficacy, making it the ideal choice for complete dentures [36,37]. Bilateral balanced occlusion minimizes the tipping forces during chewing, ensuring optimal denture retention even in patients with reduced muscular coordination. Both bilateral balanced and lingualized occlusions are especially beneficial for patients with severely resorbed alveolar ridges, as the occlusion designs compensate for compromised ridge support, ensuring better long-term retention.

Residual ridge resorption is an inevitable consequence of tooth extraction. Studies report vertical reduction rates of 11%-22% and buccolingual reductions of 29%-63% within six months post-extraction [38]. These changes significantly affect the stability of complete dentures, necessitating frequent adjustments or relining to maintain proper fit. A clinical trial conducted by Matsumaru investigated the influence of mandibular ridge resorption on masticatory efficiency in complete dentures fabricated with lingualized occlusion and bilateral balanced occlusion. The study concluded that lingualized occlusion provided superior masticatory efficiency and was the preferred occlusal scheme for patients with severely resorbed ridges [39]. This finding underscores the importance of selecting an occlusal scheme that adapts to anatomical changes over time, improving patient outcomes. Additionally, regular follow-ups are essential to monitor ridge resorption and ensure that the occlusal scheme effectively maintains function and comfort.

Removable Partial Dentures

Similar to fixed prosthodontics, occlusion in removable prosthodontics can be managed using either a conformative approach or a reorganized approach [27]. The conformative approach aims to preserve the existing occlusal relationships and harmoniously integrate the restorations with the remaining natural dentition. In contrast, the reorganized approach seeks to restore or modify the occlusion, typically to achieve a more stable centric relation (CR) or correct unfavorable intercuspation. The process typically begins with essential clinical steps, including a diagnostic cast, facebow transfer, and recording of interocclusal relations, which can be obtained in either the centric relation position or maximum intercuspal position, depending on the planned approach [40]. The conformative approach is ideal for cases where the patient’s existing occlusion is stable and functional, especially in minor edentulous areas. Conversely, the reorganized approach is preferred in cases with extensive posterior edentulous spaces, as seen in Kennedy class I or II, where stabilizing a centric relation is critical for functional support [40]. Proper planning at this stage will minimize post-placement adjustments and ensure a comfortable prosthesis for the patient.

Several occlusal schemes are available for removable partial dentures, each offering unique advantages based on the clinical situation. These include balanced articulations, neutrocentric occlusion, unilateral balanced articulation, mutually protected articulation, canine-protected articulation, and occlusion where maximum intercuspation coincides with a centric relation or a long centric [40]. Balanced articulations are generally favored for bilateral extensions, as they provide simultaneous bilateral contacts during function and enhance stability. Mutually protected articulation and canine-protected articulation are used when natural teeth coexist with the prosthesis to protect vulnerable posterior areas during excursive movements.

The choice of denture teeth materials significantly impacts occlusion stability and denture longevity, as materials vary in wear resistance, esthetics, and adaptability, influencing overall comfort, function, and preservation of vertical dimension. Acrylic resin teeth, for instance, offer resilience, wear resistance, and ease of adjustment but require frequent monitoring due to their rapid wear when opposing harder materials. Porcelain teeth, while highly durable and compatible with fixed porcelains, may cause wear on natural teeth if not properly glazed. Additionally, cast metal and ceramic surfaces, although durable and customizable to fit any occlusal scheme, require careful consideration due to cost, technical complexity, and potential esthetic limitations [40].

The positioning of artificial teeth is critical to achieving functional, esthetic, and stable outcomes. Ivanhoe emphasized that artificial teeth should ideally occupy the same anatomical positions as the missing natural teeth, with anatomical landmarks aiding in accurate positioning [40]. For anterior teeth, 3.5 mm of the incisal edge should be visible when the lips are in the rest position, helping maintain natural esthetics [41]. Additionally, the production of F and V sounds can be used to position the maxillary anterior teeth, with the incisal edge ideally falling at the wet-dry line of the lower lip (the junction between the transitional epithelium and oral mucosa) [41,42]. Proper positioning ensures both natural appearance and optimal phonetic function.

For the mandibular anterior teeth, Pound recommended using the S position as a reference. He suggested maintaining 1-1.5 mm of space between the incisal edges of the mandibular anterior teeth and the coronal surface of the maxillary anterior teeth to allow proper articulation of the S sound [42]. This spacing prevents contact during speaking, ensuring phonetic accuracy and patient comfort.

The plane of occlusion is also crucial for both function and esthetics. Ivanhoe noted that when the occlusal plane needs to be re-established, especially in Kennedy class I and II cases, a line drawn from the incisal edge of the distal-most natural teeth to the middle or upper third of the retromolar pads serves as a reliable guide [40]. Properly aligning the occlusal plane will distribute forces evenly, ensuring long-term stability and preventing denture dislodgement during function.

For posterior teeth, the occlusal plane serves as a reference for their vertical positioning, particularly in cases involving distal extensions. The placement of posterior teeth should carefully address the patient’s esthetic, phonetic, and functional needs. In situations where the ridge position and support are compromised, preference is often given to the mandibular arch to enhance denture stability [43]. A functionally generated pathway can also be employed to create a customized occlusal plane based on the patient’s mandibular movements. Techniques such as Paterson’s method provide guidance for establishing an occlusal plane in patients with posterior edentulism, ensuring alignment with natural chewing patterns and minimizing post-placement adjustments [43].

When a removable partial denture opposes a complete denture, a balanced occlusion is generally recommended to ensure optimal stability and functionality. A balanced occlusion distributes occlusal forces across the dentition, reducing tipping and enhancing prosthetic retention. This scheme is especially beneficial for patients with compromised residual ridges, where achieving stability is challenging. Additionally, the alignment of contacts during excursive movements ensures comfort and reduces stress on both prostheses, preventing premature wear or failure.

Occlusal considerations in implant-supported prosthesis

By transforming the management of partially and fully edentulous patients, the introduction of osseointegrated implants in the 1980s significantly enhanced the applicability and outcomes of restorative treatments [44]. Implant attachments stabilize dentures and can serve as abutments alongside natural teeth or as standalone supports for fixed prostheses [44]. However, developing an appropriate occlusal scheme remains essential to ensure the long-term success of both the implant and the prosthetic restoration, as implant failures often result from mechanical stress rather than biological rejection [44-46].

Implant-supported restorations are subjected to both functional loads such as chewing and parafunctional loads such as bruxism; these can generate prolonged and excessive stress [45]. Unlike natural teeth, implants lack a periodontal ligament to act as a cushion and provide proprioceptive feedback. Without a periodontal ligament, the implant is not able to dynamically accommodate occlusal forces. The occlusal forces are then transmitted directly to the surrounding bone, altering the biomechanics of load distribution [46]. This direct load transfer increases stress on the crestal bone and may result in complications such as marginal bone loss, implant fractures, and prosthetic failures. Therefore, establishing an optimal occlusal scheme that properly manages occlusal loads is vital for maintaining long-term stability, preventing biomechanical failures, minimizing bone remodeling, and preserving the bone-implant interface [45,46].

Mechanical Complications, Occlusal Planning, and Recommended Schemes

Several studies emphasize the importance of proper occlusal planning for the success of osseointegrated implants [47,48]. Stilwell [49] identified common mechanical complications, including ceramic chipping (20.31%), occlusal screw loosening (2.57%), abutment and screw loosening (5.3%), and loss of cement retention (2.06%) [50]. These mechanical failures highlight the need to reduce occlusal loads and ensure the distribution of forces across the entire prosthesis. Implementing a well-designed occlusal scheme minimizes the risk of stress-related failures, which will in turn promote long-term functional stability and reduce complications.

The recommended occlusal scheme for implant-supported prostheses includes mutually protected occlusion, with canine guidance during lateral excursions and anterior guidance during protrusion. This occlusal scheme reduces the risk of excessive loading for individual implants, especially those positioned in the posterior region. Bilaterally distributed forces that place light to medium occlusal loads on adjacent natural teeth further protect implants, particularly when natural teeth and implants coexist within the same arch [51,52]. This configuration minimizes the risk of overloading either natural teeth or implants, ensuring optimal functional harmony. As implants lack the adaptive flexibility provided by the periodontal ligament in natural teeth, it is essential to avoid both working and non-working contacts. This strategy reduces shear stresses at the bone-implant interface, which could otherwise lead to implant failure [53].

It is equally important to eliminate premature contacts, as localized overloading can result in micro-damage at the bone-implant junction and compromise stability over time. Additionally, ensuring freedom in centric occlusion, also known as long centric, allows for minor discrepancies in contact without transmitting excessive forces to the implant [54]. Minimizing cantilevers is another critical factor, as lateral forces generated by cantilevers can increase the risk of stress-induced mechanical failures and further complicate prosthetic outcomes [54]. Occlusal adjustments following the placement of prostheses are often needed to modify these forces and prevent post-restorative complications.

Managing Occlusal Forces and Parafunctional Habits

Parafunctional habits, such as bruxism, present significant challenges due to the excessive and prolonged occlusal forces they exert on implants. These forces not only affect implant-supported prostheses but also compromise peri-implant bone health by promoting bone resorption. Although research is divided on whether bruxism directly causes implant failure, the use of occlusal splints is an effective strategy to evenly distribute occlusal forces across natural teeth and implants, reducing the risk of wear and structural damage [55,56].

Moreover, occlusal overload has been identified as a significant factor in mechanical complications. Chronic overload, combined with inflammation, accelerates bone loss and increases the complexity of peri-implant management. While healthy peri-implant bone can generally adapt to normal occlusal forces, uncontrolled loading, particularly in the presence of inflammation, can accelerate bone resorption and lead to peri-implant disease. Although the direct relationship between occlusal overload and peri-implantitis remains debated, excessive forces are known to exacerbate bone loss in the presence of inflammation [55]. Careful monitoring of occlusal contacts and timely intervention are key to preventing these complications. Implementing occlusal splints and regularly monitoring occlusion can minimize these risks and ensure long-term implant stability.

Influence of Prosthetic Materials and Loading Protocols on Implant Success

The choice of prosthetic materials plays a crucial role in determining how occlusal forces are transmitted to the surrounding bone. Rigid materials, such as zirconia, are highly resistant to wear but can transfer greater forces to the bone and increase the risk of overload. Conversely, softer materials such as acrylics cushion occlusal forces but are more prone to wear over time, which could affect the longevity of the restoration [56]. Therefore, careful selection of materials is needed to achieve both durability and biomechanical safety, supporting the patient’s functional and esthetic needs.

In addition to material selection, loading protocols play a critical role in ensuring implant success. Occlusal management must account for the patient’s bone quality, as poor bone density increases the risk of implant failure under stress. In cases involving type IV bone, progressive loading protocols are recommended to allow gradual force adaptation during the early stages of osseointegration [55,56]. This gradual loading reduces early implant failures and supports healthy bone integration. Adjusting the crown-to-implant ratio is another method of reducing mechanical stress on the implant. This adjustment ensures that the implant-supported prosthesis mimics natural force dynamics as closely as possible. Overall, regular monitoring and occlusal adjustments are essential for extending implant longevity and minimizing complications. This is particularly true in patients with parafunctional habits or restorations involving mixed dentition [55,56].

Occlusal considerations in adhesive restorations

A conformative approach is typically adopted in cases involving adhesive or bonded restorations unless extensive occlusal corrections or full-mouth rehabilitation using adhesive restorations are required [57]. This approach preserves the patient’s existing occlusal scheme while ensuring that the restoration integrates into the dentition.

In a comprehensive two-part article on occlusion, Warreth et al. summarized several key factors that should be considered before initiating a tooth surface restoration [58]. First, it is essential to verify the maximum intercuspal position (MIP) before commencing any tooth preparation. This ensures that the restoration will align properly with the existing occlusal contacts. Any occlusal interferences should be corrected before starting the restorative work to prevent potential complications, such as premature wear or fracture of the restoration.

In addition, the junction between the restoration and sound tooth structure should, wherever possible, be free from contact with the working cusp to reduce the risk of stress concentration in these areas. For porcelain-fused-to-metal restorations, it is critical to maintain a 2 mm clearance from the opposing contact in the maximum intercuspal position [57,58]. Material properties, such as hardness and fracture toughness, are essential in fixed prosthodontics to withstand the variety of occlusal forces exerted during function. Porcelain, for instance, provides excellent esthetics but is prone to fracture under heavy occlusal loads, whereas metals and high-strength ceramics such as zirconia offer enhanced durability. Selecting materials with adequate strength properties helps mitigate the risk of restoration failure in high-stress regions [57-59]. Furthermore, placing occlusal contact on metal is generally preferable to porcelain, as metal offers superior resistance to occlusal forces [57,58].

When zirconia is used, the substructure should be extended incisally or occlusally to provide support to the ceramic veneer at the incisal edge and cusp, respectively. This reinforcement helps distribute occlusal loads more evenly and minimizes the risk of chipping or delamination of the veneer. For cantilever restorations with a single pontic, care should be taken to ensure that the pontic is kept out of contact during all excursive mandibular movements to prevent shear stresses from damaging the restoration [57,58].

A custom-made incisal guide may be needed when the palatal surfaces of the upper anterior teeth are involved in the new restoration. This guide helps ensure proper guidance during functional movements, reducing the likelihood of interferences [58-60]. For patients with parafunctional habits, such as bruxism, the use of an occlusal splint following restoration is recommended to protect the newly restored surfaces from excessive forces. Regular monitoring of splint fit and function is essential to ensure ongoing protection against parafunctional habits.

A comprehensive functional assessment of both extraoral and intraoral structures is required both before and after completing the restorative treatment [28,59-61]. The extraoral examination should include an evaluation for signs of muscular hyperactivity, such as muscle tenderness or hypertrophy, as well as symptoms of temporomandibular disorders (TMDs), including pain, clicking, or deviation during mandibular movements [60].

Intraoral examinations should assess the maximum intercuspal position for any mismatch between centric relation (CR) and maximum intercuspal position. The presence of long centric, arc of closure, and line of closure should also be evaluated, as these factors can impact the long-term success of the restoration. Additionally, the clinician should be alert for signs of parafunctional habits or trauma from occlusion, which may manifest in both hard and soft tissues [60]. Detecting and managing these signs early will help avoid restoration failure, maintain patient comfort, and prevent complications that could affect the longevity of the restoration [59,60]. Table 1 shows occlusal considerations and recommendations across restoration types.

**Table 1:** Occlusal Considerations and Clinical Recommendations for Various Restoration Types RPDs: removable partial dentures

Restoration Type	Occlusal Considerations	Key Features	Clinical Recommendations
Types of Restorations	Occlusion is critical for all types of restorations (e.g., crowns and implant-supported prostheses). Premature contacts and occlusal balance must be managed to avoid complications [14,15].	Includes both tooth and implant-supported restorations, with different occlusal load distributions; absence of PDL in implants increases bone stress [16].	Perform thorough pre-treatment assessments, check for premature contacts, and ensure stress distribution through occlusal adjustments [17-20].
Fixed Prosthodontics	Mutually protected occlusion and group function occlusion are key schemes [21]. The approach depends on whether occlusal corrections are minor (conformative) or major (reorganized) [27].	In group function, multiple teeth guide lateral movements; in canine guidance, only the canines do [21]. Reorganized approach involves centric relation and diagnostic wax-ups.	Use group function for multiple contacts and canine guidance for reducing posterior stress. Opt for a reorganized approach when significant occlusal changes are needed.
Complete Dentures	Different schemes like neurocentric, lingualized, and balanced occlusion are used [32]. Lingualized and balanced occlusions are ideal for patients with severe ridge resorption [39].	Neurocentric has flat occlusal surfaces; lingualized occlusion focuses on maxillary palatal cusps; balanced occlusion distributes forces evenly across all teeth during movement [33-37].	For resorbed ridges, use lingualized or balanced occlusion. Ensure proper tooth positioning based on the occlusal plane for stability and esthetics [39].
RPDs	Common schemes include balanced, neutrocentric, and mutually protected occlusion. Teeth positioning must align with anatomical landmarks, with careful attention to the occlusal plane [40].	Balanced occlusion is often used for RPDs that oppose complete dentures. Neutrocentric occlusion has flat surfaces, allowing for freedom of movement without interference [40].	Ensure proper occlusal plane, especially in distal extensions. Use balanced occlusion when opposing complete dentures for stability [40].
Adhesive/Bonded Restorations	Typically follows a conformative approach unless major occlusal changes are needed [57]. Proper clearance and contact management are essential, especially for porcelain or zirconia restorations [58].	Porcelain and zirconia require careful substructure support to handle occlusal loads. For cantilever restorations, the pontic should be out of contact during excursions [58].	Perform functional assessments pre- and post-restoration. In bruxism patients, use occlusal splints. Ensure that occlusal forces are distributed across the restoration to prevent mechanical failure [59,60].

Effects of occlusion on restoration longevity

Numerous factors influence the longevity of dental restorations, including the size of the restoration, the tooth’s position within the arch, the patient’s caries index, material selection, moisture control, and various other patient- and clinician-related factors [61]. Among these, occlusion remains one of the most significant yet often overlooked factors contributing to restoration failure. A lack of understanding of basic occlusal principles can result in ineffective trial-and-error approaches to addressing patients’ complaints and ultimately compromise the outcomes of the restoration [62]. This underscores the importance of proper occlusal analysis during both the planning and execution phases of restorative procedures.

Several studies have evaluated the effects of occlusion on the durability of restorations. Restorations conforming to the existing dentition show better longevity compared to those with high occlusion or interferences during excursive movements, where the restoration experiences unfavorable forces [63]. Therefore, it is essential to ensure that the restorations align with the patient’s natural occlusal scheme to minimize stress and prevent premature failure. Conversely, restorations with improper occlusal contacts can lead to localized stress concentration, resulting in microcracks or debonding over time.

Material selection plays a crucial role in how restorations withstand occlusal loads. Amalgam has long been favored for patients with parafunctional habits or heavy occlusal forces, as well as in cases where the occlusal load falls directly on the restoration [64]. Its durability under stress makes it a reliable option for these situations. However, with advancements in material science, composite resin has emerged as a promising alternative for posterior restorations, offering strong bonding capabilities and the ability to bear occlusal loads during function [65]. Heintze and Rousson conducted a meta-analysis on posterior resin restorations and reported a 5% failure rate due to material fracture and approximately 12% noticeable wear over 10 years [66].

When the occlusal load exceeds the material’s capacity, the restoration becomes prone to cracking and fracturing [67]. Multi-surface restorations, such as class II or class IV restorations, are particularly susceptible to fractures due to their larger surface areas and complex occlusal relationships [68]. When restoring multiple surfaces, not only does material choice play a role, but it is also crucial to achieve proper occlusal harmony to reduce stress at contact points. In a clinical study, Özcan and Niedermeier examined failures in metal-ceramic restorations and found that 65% of failures occurred in the anterior region, with 60% on the labial surface, 27% on the buccal, 5% on the incisal, and 8% on the occlusal surfaces [69]. Most fractures were observed in the maxillary arch (75%), particularly on the labial surfaces of anterior teeth.

Long-term evaluations of ceramic restorations indicate high survival rates, with a success rate of 97.3% at five years, 93.5% at 10 years, and 78.5% at 20 years, based on a clinical evaluation of 1,335 all-ceramic restorations [70]. While porcelain has significant esthetic advantages as an indirect restorative material, its brittle nature limits its durability. Recent advancements in dental ceramics have improved the compressive strength of these materials, enhancing their ability to withstand occlusal forces and tolerate shear stresses more effectively than earlier generations of porcelain [71]. These innovations have made ceramic restorations more reliable by offering both functional and esthetic benefits.

Occlusal trauma and its effects on restorations

Occlusal trauma, or trauma from occlusion, refers to injury to the attachment apparatus that results from excessive occlusal force [72]. Periodontal health is closely linked to the forces applied to teeth. While moderate occlusal forces can enhance periodontal health by thickening the lamina dura and reorganizing bony trabeculae, excessive forces can overwhelm these adaptive mechanisms and cause tissue injury. This tissue damage can present as increased tooth mobility, widened periodontal ligament spaces, and bone resorption.

Before initiating any restorative treatment, a thorough clinical examination is essential to ensure functional balance in the masticatory system. This examination should include an evaluation of the orthopedically stable joint position, functional tooth position, the direction and magnitude of occlusal forces, and signs of occlusal overload [73]. Identifying occlusal overload early can prevent potential complications, such as fractures in restorations or further damage to the periodontium. In addition, special attention is needed when restoring anterior teeth to identify excursive contacts and high points during maximum intercuspation. While functional loads can support osseointegration in implants, excessive occlusal loads may lead to implant failure [73]. As implants lack the adaptive properties of the periodontal ligament, they are particularly susceptible to damage under excessive forces.

When a complete denture opposes a partial denture, a balanced occlusal scheme is recommended to maintain optimal function and stability [54,74]. This balance minimizes tipping forces and ensures even force distribution across the prostheses. Occlusal assessments using articulating paper or shimstock are essential, and occlusal equilibration may be necessary to eliminate high points [74]. Regular follow-ups to monitor the occlusion are needed to address any developing imbalances that may arise over time.

The Role of Parafunctional Habits in Restoration Longevity

The masticatory system’s occluding surfaces come into contact during both functional and parafunctional activities. Functional activities include eating and speaking, while parafunctional activities are clenching, grinding, nail-biting, and similar habits [75]. Parafunctional habits are common, with Melchior et al. reporting that 73% of participants engaged in teeth clenching and grinding [76].

Parafunctional habits can cause significant damage to the dentition and prosthetic restorations, leading to enamel wear, tooth fractures, and soft tissue changes [77]. These habits also negatively impact prostheses and contribute to failures in composite resin restorations, crowns, bridges, implants, and removable prostheses. Damage to prosthetic materials, such as microfractures and debonding, can accumulate over time and increase the need for repair or replacement. Despite considerable research, the underlying causes of parafunctional habits are not yet fully understood. Some studies suggest that psychosocial factors and hormonal imbalances, such as irregularities in dopamine and serotonin levels, may influence these behaviors [77-79]. Stress, anxiety, and sleep disorders are also closely associated with the development and persistence of bruxism, indicating the multifactorial nature of parafunctional habits.

As a protective measure to safeguard restorations, occlusal splints are commonly recommended for patients with parafunctional habits. The splints help distribute occlusal forces evenly across the dental arches, minimizing the impact on specific teeth or restorations. Hachmann et al. demonstrated that children with bruxism who were fitted with occlusal splints experienced no increase in wear facet size compared to a control group [80]. Takahashi et al. further investigated the effectiveness of palatal and stabilization splints in reducing masseter muscle activity in bruxism patients and observed a significant reduction in muscle activity with splint use [81]. Landry et al. found that an adjustable mandibular advancement appliance (MAA) was more effective than occlusal splints in managing sleep bruxism, significantly reducing the frequency of bruxism episodes per hour [82]. The study emphasized that customized appliances tailored to individual patient needs yielded better outcomes in managing nocturnal bruxism. However, while occlusal splints provide valuable protection for restorations and dentition, they should not be used as a way of treating underlying parafunctional habits [79-82]. Long-term management of these habits involving a multidisciplinary approach is required, combining behavioral therapy, pharmacological interventions, and stress management techniques. Table 2 shows occlusal factors impacting restoration longevity.

**Table 2:** Occlusal Factors Influencing Restoration Longevity and Failure

Aspect	Description	Key Features/Factors	Limitations/Challenges
Impact of Occlusion on Restoration Longevity	Occlusion plays a significant role in determining the long-term success or failure of restorations. Poorly managed occlusal forces can lead to material fractures, wear, and eventual restoration failure [62].	Improper occlusion can lead to cracking, fracturing, or failure in multi-surface restorations (e.g., class II or IV), especially under heavy occlusal loads [68].	Failure to manage occlusion results in excessive stress on the restorations, causing premature wear or fractures, especially in the case of large or complex restorations [68].
Occlusal Trauma and Its Effect on Restorations	Excessive occlusal forces can cause trauma to the periodontium and lead to premature failure of restorations, especially implant-supported restorations [73].	Implants lack periodontal ligaments, so occlusal loads are transferred directly to the surrounding bone, increasing the risk of bone loss, implant fracture, and restoration failure [73].	Excessive forces can result in irreversible damage to the surrounding structures (e.g., implants and TMJ), and managing occlusal forces in these cases can be challenging [73,74].
Parafunctional Habits and Their Impact	Parafunctional habits, such as bruxism, can severely affect the longevity of restorations by causing wear, fractures, or damage to both natural teeth and prosthetics [77].	Splints are commonly used to protect restorations from excessive forces generated by parafunctional habits, helping to distribute forces more evenly across the dentition [80].	Splints do not address the underlying cause of parafunctional habits (e.g., bruxism), and long-term use can be uncomfortable for patients [80-82].
Material Considerations	The choice of restorative material influences the restoration’s ability to withstand occlusal forces. Some materials, such as amalgam, can handle heavy occlusal loads better than others, such as composites or ceramics [64].	Amalgam is preferred for patients with high occlusal forces or parafunctional habits, while composite resins show higher failure rates in posterior restorations under heavy loads [65].	Composite resins and ceramics, although esthetically appealing, are more prone to fractures and wear under heavy occlusal forces, especially in multi-surface restorations [65,66].
Failure of Restorations Due to Occlusion	Common restoration failures include fracture, chipping, loosening, or wear, particularly in posterior regions where occlusal forces are highest [49].	Metal-ceramic restorations tend to fail in anterior regions, with failure rates varying depending on factors such as material choice and occlusal load distribution [69].	Without proper occlusal management, restorations such as implants, bridges, and crowns are prone to mechanical failure [49,67]. (e.g., screw loosening, chipping, and fractures).
Role of Splints in Managing Occlusal Forces	Occlusal splints are used to mitigate the effects of occlusal overload, especially in patients with parafunctional habits such as bruxism. They help reduce the risk of damage to restorations [55,56].	Splints reduce wear on restorations and distribute forces more evenly. Studies have shown a significant reduction in masseter muscle activity and wear facet size with splint use [81].	Splints do not address the underlying cause of parafunctional habits and are not a permanent solution [80-82]. Some patients may find splints uncomfortable for long-term use.

Effects of occlusion on TMJ

Temporomandibular disorders (TMDs) are one of the most prevalent causes of orofacial pain and affect approximately 5%-⁠12% of the population [83,84]. The role of occlusion in TMD has been widely debated. Lipp examined the relationship between temporomandibular disorders and occlusion, concluding that experimental, epidemiological, and clinical studies do not provide strong evidence supporting the role of occlusion in TMD development. Lipp suggested that the articulatory system’s capacity for remodeling enables it to adapt to most forms of occlusal function and dysfunction [85].

In a series of studies, Seligman and Pullinger identified certain occlusal characteristics associated with TMD, such as an overjet greater than 4 mm, unilateral posterior crossbite, and a slide from a retruded contact position to an intercuspal position exceeding 1.75 mm [86]. However, these associations were statistically weak, and no single occlusal factor reliably distinguished TMD patients from healthy individuals. This suggests that while occlusal factors may contribute to TMD, they are unlikely to be the sole cause of TMD.

Another study explored the role of psychosocial functioning and dental factors in adolescents with TMD compared to healthy subjects [87]. Both groups completed standardized questionnaires and underwent clinical examinations. While no significant dental differences were identified, the TMD group reported higher levels of stress, somatic complaints, fatigue, and aggressive behavior. This suggests that psychosocial factors may play a more significant role in TMD development than dental factors, particularly in adolescents. These findings align with other research suggesting that psychological stress and emotional health are critical in understanding the etiology of TMD.

Ohta et al. investigated the relationship between balancing-side occlusal contact patterns and TMJ internal derangement using MRI scans and occlusal examination in 41 patients. They found that a simultaneous balancing-side contact was associated with disc dislocation [88]. In contrast, Minagi et al. observed a significant correlation between the absence of non-working side contacts and a higher prevalence of joint sounds, indicating that non-working side contacts may serve a protective function [6]. This protective role may involve dispersing lateral forces during functional movements and thus reducing the strain placed on the TMJ.

Various systematic reviews have examined the association between occlusion and TMD, but none have found conclusive evidence of a direct relationship [89,90]. Some studies suggest that occlusal disharmony could be associated with TMD, although it may be more of a sequela than a cause [9,81]. This highlights the complex and multifactorial nature of TMD, where occlusal factors may interact with psychological and biomechanical influences. A thorough patient history and careful extraoral and intraoral examination remain essential for diagnosing TMD [9]. Given that TMDs are generally self-limiting, most orofacial pain societies recommend conservative and reversible treatments as the initial approach. These include counseling, behavioral modifications, splint therapy, pharmacotherapy, and physiotherapy [91].

More invasive treatment options, such as occlusal equilibration, orthodontic correction, and occlusal adjustments with restorations, have also been investigated by researchers. However, systematic reviews highlight the lack of sufficient randomized controlled trials to confirm the effectiveness of these treatments [92,93]. This lack of evidence has led clinicians to emphasize conservative strategies as the gold standard in managing TMD, reserving invasive procedures for cases unresponsive to less invasive therapies. As a result, further research is needed to establish a definitive cause-and-effect relationship between these treatments and TMDs.

Occlusal analysis and diagnostic tools

Achieving stable and harmonious occlusion requires careful attention to occlusal interferences, particularly excursive interferences, and undesirable tooth contacts that occur during mandibular movements. These interferences can compromise joint stability and disrupt the occlusal balance. The three main types are (1) working side interference, which disrupts movement on the side of mandibular motion; (2) non-working side interference, which shifts the fulcrum away from the TMJ, risking off-axis forces and instability; and (3) protrusive interference, causing excessive force on anterior teeth during forward movement [7,58]. Detecting and managing these interferences is essential to prevent complications, especially in complex occlusal adjustments.

Given the potential impacts of excursive interferences and other occlusal issues, accurate diagnosis is crucial to prevent unnecessary adjustments or complications following restorative treatments [94-98]. Numerous diagnostic methods have, therefore, been developed to enhance occlusion evaluation both before and after restorative procedures. These tools assist clinicians in identifying issues such as premature contact, occlusal asymmetry, and occlusal trauma, which can all negatively affect the longevity of restorations.

One early technique is occlusal sonography, introduced in Japan in the late 1980s. The Dental Sound Checker was designed to detect sounds produced by teeth during closure to identify occlusal disturbances based on principles outlined by Watt [94]. However, variability in sound production, which depends on the force applied during tooth contact, limited the method’s effectiveness and reliability, leading to the search for more precise and quantifiable methods.

The T-Scan system marked a significant advancement in occlusal diagnostics. This computerized tool uses a pressure-sensitive film to record and display individual occlusal contact pressures. The latest version, T-Scan 5, employs a color-coded system to represent varying levels of occlusal force, with blue indicating optimal force and red indicating high force. Widely used to diagnose occlusal trauma, premature contacts, and force asymmetry, especially in patients with temporomandibular disorders, the T-Scan also plays a vital role in occlusal equilibration during restorative and orthodontic treatments. By providing a dynamic view of occlusal interactions, the T-Scan helps clinicians fine-tune restorations, improving patient comfort and minimizing post-treatment complications. However, the sensor thickness of 0.1 mm, which is thicker than traditional tools such as articulating paper and shimstock, can affect the accuracy of occlusal recordings [95].

A more recent advancement is the OccluSense device, which was introduced in 2019. This wireless system transmits occlusal data via Wi-Fi to an iPad application, providing real-time displays of occlusal forces. With a sensor thickness of 60 microns, OccluSense offers improved tactile sensitivity over the T-Scan, allowing clinicians to capture more subtle force interactions for precision dentistry. However, despite its advanced features, it requires daily function tests to ensure accuracy. Additionally, its reliability, repeatability, and validity are not as well-supported scientifically as the T-Scan’s 36-year track record [96].

According to a survey by Bozhkova et al., articulating paper remains the most commonly used tool for occlusal analysis, followed by articulating silks, foils, and shimstock [97]. Additional tools include liquid occlusal indicators, such as rouge, and virtual dental patient models. Virtual models, created through intraoral scanners, allow clinicians to analyze occlusal discrepancies digitally, providing both precision and convenient storage for future reference. As digital dentistry grows, virtual models are gaining popularity for their ease of use and accuracy [97].

Articulating paper is hydrophobic and comes in various thicknesses and dye types, adhering to the tooth surface upon contact to reflect applied force. Although widely used, it has limitations: with the tactile sensitivity of natural dentition around 8-10 microns [98], the typical thickness of articulating paper (40 microns) can exceed the patient’s perception threshold, potentially causing inaccuracies in detecting subtle discrepancies.

In contrast, shimstock offers a more accurate method of recording high points in occlusion. Available in thicknesses ranging from 8 to 12 microns, this thin metallic film provides precise detection of occlusal contacts, making it ideal for identifying discrepancies that thicker materials, such as articulating paper, might miss [97,98]. Shimstock is particularly valuable in complex cases, such as full-mouth rehabilitations, where precise occlusal adjustments are critical for long-term success. Table 3 shows a comparison of tools and devices for occlusal analysis.

**Table 3:** Comparison of Tools and Devices for Occlusal Analysis: Features and Limitations

Device/Tool	Description	Key Features	Limitations
Occlusal Sonography [94]	The “Dental Sound Checker,” introduced in Japan in the late 1980s, detects teeth contact sounds to identify occlusal disturbances.	Detects occlusal disturbances based on sound during closure.	Sound variation depending on closure force limits accuracy.
T-Scan [95]	T-Scan is the first computerized occlusal analysis system, developed in 1987. The current version, T-Scan 5, analyzes and displays occlusal contact pressures using color-coded columns.	Uses color range from blue to red to represent occlusal forces; applied for diagnosing occlusal trauma and detecting premature contact and occlusal equilibration in treatments.	Film thickness of 0.1 mm, thicker than articulating paper and shimstock, limiting precision.
OccluSense [96]	OccluSense, a wireless occlusal indicator device that transmits occlusal force data to an iPad app via Wi-Fi, was introduced in 2019.	60-micron thick sensor, wireless, data visualized on app; requires daily function tests to ensure proper working.	Lacks scientific evidence for reliability, repeatability, and accuracy compared to T-Scan’s 36-year history.
Articulating Paper Strips and Shimstock [96,99]	They are commonly used for occlusal analysis. Articulating papers leave dye marks on occlusal contact points. Shimstock is a metallic film used to detect occlusal high points.	Articulating paper is available in various thicknesses; it leaves dye marks based on occlusal force. Shimstock (8-12 microns thick) offers more accurate occlusal high-point detection.	Articulating paper thickness (40 microns) exceeds patients’ occlusal tactile sensitivity (8-10 microns), leading to inaccuracies.

## Conclusions

Achieving and maintaining optimal occlusion is crucial for the long-term success of dental restorations, impacting both their functional stability and patient satisfaction. Occlusal harmony not only ensures an even distribution of forces but also minimizes complications such as fractures, wear, and occlusal trauma. Various restorative modalities, including fixed and removable prostheses, implant-supported restorations, and adhesive restorations, require tailored occlusal approaches to accommodate individual clinical needs. Additionally, the integration of advanced diagnostic tools, such as T-Scan and OccluSense, enhances the precision of occlusal analysis, helping clinicians detect and address discrepancies early. While occlusion alone may not directly cause conditions such as TMD, it plays a contributory role and should be carefully managed in comprehensive treatment plans. Furthermore, managing parafunctional habits through occlusal splints and regular follow-ups is essential in preserving both natural dentition and restorations. Ultimately, a thorough understanding of occlusal principles is indispensable for clinicians to achieve successful restorative outcomes, enhance patient comfort, and ensure the longevity of dental restorations.
